# Food Allergy-Related Bullying in Pediatric Patients: A Systematic Review

**DOI:** 10.3390/children11121485

**Published:** 2024-12-05

**Authors:** Rita Nocerino, Caterina Mercuri, Vincenzo Bosco, Greta Aquilone, Assunta Guillari, Silvio Simeone, Teresa Rea

**Affiliations:** 1 Department of Translational Medical Science, University of Naples Federico II, 80131 Naples, Italy; gr.aquilone@studenti.unina.it (G.A.); assunta.guillari@unina.it (A.G.); 2 ImmunoNutritionLab at CEINGE Advanced Biotechnologies, University of Naples Federico II, 80131 Naples, Italy; 3 Department of Biomedicine and Prevention, University of Rome “Tor Vergata”, 00133 Rome, Italy; 4 Department of Clinical and Experimental Medicine, University of Catanzaro MagnaGraecia, 88100 Catanzaro, Italy; c.mercuri@unicz.it (C.M.); vincenzo.bosco@unicz.it (V.B.); silvio.simeone@unicz.it (S.S.); 5 Department of Public Health, University of Naples Federico II, 80131 Naples, Italy; teresa.rea@unina.it

**Keywords:** food allergy, bullying, pediatric patients, systematic review, mental health, school interventions

## Abstract

Background: Food allergy (FA)-related bullying is a significant public health concern affecting pediatric patients. This systematic review investigates the prevalence, characteristics, and psychosocial impact of FA-related bullying, as well as current intervention strategies within educational and healthcare settings. Methods: A systematic literature search was conducted across the PubMed, Web of Science, and CINAHL databases, covering publications up to February 2024. The review followed PRISMA guidelines and included studies on children and adolescents (0–18 years) diagnosed with FAs. Studies were selected based on eligibility criteria and assessed for quality using the Newcastle–Ottawa Scale. Results: The initial search identified a total of 260 records (6 from scientific databases and 254 from registries). Twenty-six studies met the inclusion criteria. The findings of these studies reveal that FA-related bullying is prevalent, with rates varying between 17% and 60%, depending on the study population and methods. Bullying often involves verbal teasing, social exclusion, and physical threats using allergens, presenting both psychological and physical risks. Psychological consequences include increased anxiety, depression, and social withdrawal, which persist over time, significantly impacting quality of life for both children and their families. Notably, bullying often occurs in school settings, emphasizing the need for targeted interventions. Conclusion: FA-related bullying profoundly affects mental health and quality of life for affected children and their families. Interventions, such as school-based allergy education programs and policies promoting inclusivity and safety, have shown promise in reducing bullying incidents. A collaborative approach involving healthcare providers, educators, and policymakers is essential to mitigate the impact of FA-related bullying and improve outcomes for affected children.

## 1. Introduction

Food allergies (FAs) are characterized as adverse health effects stemming from specific immune responses triggered by the ingestion of certain foods [1]. This abnormal immune reaction is primarily due to a breakdown in immune tolerance mechanisms, influenced by multifaceted interactions between genetic, epigenetic, and environmental factors [2,3].

FAs arise when the immune system erroneously perceives harmless food proteins—commonly present in cow’s milk, eggs, nuts, fish, and soy—as dangerous, triggering an immune response that can vary from mild symptoms, such as skin rashes, to severe and potentially life-threatening anaphylaxis [1]. Recent research highlights how alterations in microbiome composition can weaken immune tolerance and promote allergic responses, illustrating the crucial role of the gut microbiome in FA development [4].

The prevalence of FAs has been steadily increasing worldwide, especially among pediatric populations [5]. FAs now affect up to 10% of children in high-income countries, showing a significant upward trend in recent years [5,6]. A similar increase has also been observed in newly industrialized countries, highlighting the global rise in FA prevalence [7,8]. This rise has led to increased hospital admissions for severe allergic reactions, underscoring the need for public health strategies focusing on prevention, early diagnosis, and management [9,10].

The daily impact of FAs extends far beyond dietary restrictions, disrupting various aspects of a child’s life and social interactions, particularly through exclusion from group activities [11], isolation [12], and discrimination [13]. Children with FAs face a constant threat of accidental allergen exposure, necessitating careful avoidance strategies that can limit participation in school events, social gatherings, and cultural practices involving shared meals [14]. This constant risk contributes to anxiety, stress, and a diminished quality of life (QoL) for both children and their families [15]. Furthermore a particularly distressing aspect of FAs is the risk of anaphylaxis, a severe, rapid-onset reaction that can be fatal without immediate intervention [16]. Families are often required to maintain constant vigilance and carry emergency medications, which places considerable psychological strain on both children and their caregivers [16].

All this entails that children with FAs face social challenges, such as stigma and bullying, exacerbating their psychological burden [14].

The US Centers for Disease Control and Prevention defines bullying as “any unwanted aggressive behavior(s) … that involves an observed or perceived power imbalance and is repeated multiple times or is highly likely to be repeated” [17].

In children with FAs, bullying often involves verbal teasing, social exclusion, mocking food restrictions, and even deliberate threats or attempts to expose them to allergens [18]. Such bullying behaviors pose both psychological and physical risks, contributing to increased anxiety, depression, social withdrawal, post-traumatic stress disorder, decreased self-esteem, and school avoidance, as well as long-term impacts on QoL for both children and their families [19,20,21]. This type of bullying not only disrupts the child’s mental health and social development, but also introduces serious health dangers, making it a significant concern for those affected [19].

Therefore, the primary objective of this systematic review is to analyze the prevalence of bullying among children with FAs, examining demographic factors such as age, gender, and allergy severity to identify vulnerable groups and provide guidance for targeted interventions.

A further objective is to assess the psychological and social impacts of bullying on children with FAs.

Finally, this review aims to examine current strategies to manage and prevent FA-related bullying.

## 2. Materials and Methods

### 2.1. Search Strategy and Compliance with Ethics Guidelines

This systematic review was performed in accordance with the 2020 PRISMA guidelines [22]. A comprehensive search was conducted in major scientific databases, including PubMed, Web of Science, and CINAHL, covering all publications up to 29 February 2024. This systematic review has been registered in the PROSPERO database (ID CRD42024510180). Search terms included “food allergy bullying”, “pediatric allergy bullying”, and “psychosocial impact of allergy-related bullying” to ensure an exhaustive literature review.

### 2.2. Eligibility Criteria

Articles eligible for inclusion in this review had to meet the following inclusion criteria: studies involving children and adolescents aged 0–18 years with an FA diagnosis; studies that included patients with FAs who completed a questionnaire on bullying; comparative studies with control groups of children without FAs, allowing the prevalence and types of bullying in both groups to be examined and compared; articles published in English. All article abstracts were screened by three authors (R.N., G.A., and C.M.) working in pairs in a blinded fashion; those that did not meet the inclusion criteria were excluded; any controversies were resolved by consensus in a meeting in which the abstracts were reviewed.

Articles that met any of the following criteria were excluded from the review: studies not involving a diagnosed FA or not specifically targeting children and adolescents aged 0–18 years; articles lacking a control group for comparative analysis if relevant to the study design; studies not published in English; conference abstracts, letters, editorials or other non-peer-reviewed contents; studies that were duplicates, incomplete or had insufficient data for analysis.

Articles that met the exclusion criteria were omitted from the review. Three authors (R.N., G.A., and C.M.) independently screened all article abstracts in pairs under blinded conditions. Articles failing to meet the inclusion criteria were excluded, and any discrepancies were resolved through consensus during a meeting where the abstracts were reassessed.

### 2.3. Quality Assessment

All observational studies were evaluated using the Newcastle–Ottawa Scale (NOS) and its adapted versions, designed to assess the quality of non-randomized cross-sectional, case–control, and cohort studies. The scale assigns a maximum of 10 stars, focusing on selection (including representativeness, sample size, non-respondents, and exposure ascertainment), comparability, and outcome assessment (such as evaluation methods and statistical testing) [23,24].

## 3. Results

### 3.1. Eligible Studies

**Figure 1:**
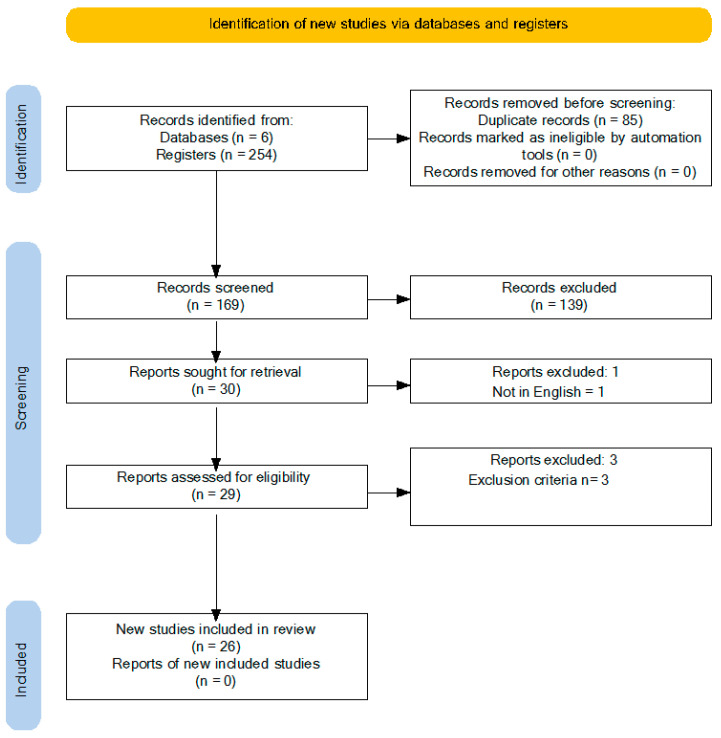
PRISMA diagram.

The initial search identified a total of 260 records (6 from scientific databases and 254 from registries). After eliminating 85 duplicates, 169 records were screened. Of these, 139 were excluded based on the title and abstract, leaving 30 articles for full-text review. One non-English article was excluded, resulting in twenty-nine articles being suitable for eligibility assessment. After further evaluation, additional articles were excluded for not meeting the inclusion criteria (e.g., studies not specific to FAs or FA-related bullying). Ultimately, 26 studies met the inclusion criteria and were included in the systematic review. The authors reached complete agreement (100%) on the inclusion and exclusion of articles following a discussion where each article was assessed based on the inclusion criteria. A PRISMA flow chart outlining the sequential steps in the study selection process is shown in Figure 1.

### 3.2. Quality Assessment of the Reviewed Studies

One study was not scored because of its qualitative study design. The quality of the remaining reviewed studies was evaluated using appropriate tools based on their study design (Table 1). Most studies were rated as moderate to high quality, with a minimum score of 5.

### 3.3. Prevalence of FA-Related Bullying

Prevalence rates of FA-related bullying vary widely, influenced by cultural attitudes and awareness. Across the reviewed studies, the prevalence of FA-related bullying in children was consistently high. Torabi et al. (2016) [42] reported that 20% of food-allergic children in Canada experienced bullying, with 77% of those repeatedly targeted, often at school. Similar findings were noted in the FORWARD study in the U.S., where 20% of children aged 4 to 15 reported bullying, with rates increasing to 33% in children over 11 [21]. In Australia, Fong et al. (2018) [27] found that 42% of children with FA had been bullied, with 23% specifically targeted due to their allergy. Other studies reported even higher rates; for example, Rocheleau and Rocheleau (2019) [34] found that 45–60% of children with FAs experienced bullying, and Lieberman et al. (2010) [28] documented 24%, with multiple bullying episodes occurring in 86% of those cases. The prevalence varied significantly depending on the method of assessment and country of study [25,30]. These disparities underscore the importance of culturally tailored interventions to address bullying effectively. In Table 2, the prevalence, bullying characteristics, and key interventions across different countries are summarized.

### 3.4. Characteristics of Bullying

The bullying of children with FAs primarily involves non-physical forms such as verbal teasing, social exclusion, and physical threats involving allergens. In Torabi et al.’s (2016) [42] study, 86% of bullying occurred at school, was initiated by classmates, and mainly took the form of verbal teasing and social exclusion. Abrams et al. (2020) [11] and Fong et al. (2018) [27] highlighted that bullying frequently involved food allergens, with children being threatened or harassed by others waving or throwing allergens at them. In some severe cases, children were forced to touch or even ingest allergens [34]. Physical threats involving allergens were found to be particularly traumatic for children. These incidents often occurred during school hours or at social gatherings [31].

See Table 2 for bullying characteristics across different countries.

### 3.5. Psychological and Social Impacts

The psychological impact of FA-related bullying is profound, leading to increased anxiety, depression, and diminished QoL in both children and their parents. Annunziato et al. (2014) [20] found that bullying significantly raised distress levels in children and parents, reducing their QoL. Abrams et al. (2020) [11] also documented the emotional toll on families, with parents reporting feelings of isolation and helplessness. The study by Rocheleau et al. (2022) [33] confirms that bullying contributed to anxiety, depression, and social withdrawal in children, with 65% of bullied children experiencing embarrassment and emotional distress. Moreover, the impact extended to parents, who faced increased stress and anxiety. These psychological effects frequently endured over time, potentially contributing to long-term mental health challenges [38].

### 3.6. Longitudinal Impact on Mental Health

The long-term effects of FA-related bullying are concerning, as bullying often continues over time. Annunziato et al. (2014) [20] demonstrated that 69% of children who were bullied at baseline continued to experience bullying a year later, significantly impacting their QoL. Persistent bullying was linked to chronic psychological distress, leading to anxiety and depression that could persist into adulthood [38,39].

### 3.7. Differences by Demographics

Several demographic factors, such as age, gender, and the number of FAs, influence the likelihood and impact of FA-related bullying. Fong et al. (2018) [27] found that older children and adolescents were more likely to experience bullying compared to younger children, a finding echoed by Rocheleau et al. (2022) [33]. Brown et al. (2020) [21] noted that older White children reported higher rates of bullying than their Black peers. Gender differences were also observed; girls tended to experience social exclusion, while boys were more likely to face physical bullying involving allergens [30]. Furthermore, children with multiple FAs were at higher risk of severe bullying, as highlighted in Abrams et al. (2020) [11] and Polloni et al. (2016) [31]. These demographic factors significantly shaped the nature and impact of bullying, making certain groups more vulnerable to its effects.

### 3.8. Current Strategies to Manage and Prevent FA-Related Bullying

#### 3.8.1. School-Based Interventions and Education Programs

Schools play a crucial role in addressing bullying related to FAs by implementing structured interventions and educational programs that foster safer, more inclusive environments for students with FAs. Comprehensive allergy management policies, which include targeted awareness campaigns and specific training for staff, effectively reduce bullying and enhance safety for FA-affected students [28,42]. Programs that raise awareness of FAs among students and staff while educating them on the serious consequences of allergen exposure have been shown to reduce stigma and promote empathy, which are essential for building supportive peer relationships [28].

Research from Canada supports the effectiveness of allergy-specific educational modules and mandatory training for school staff in reducing stigma and increasing student safety. These measures are particularly impactful when coupled with policies that restrict allergens in cafeterias and promote designated eating zones, fostering a positive school environment for children with FAs [11]. Muraro et al. (2014) [30] further demonstrated that integrating FA education into school curricula empowers both teachers and students to recognize and respond to bullying, reinforcing policies and promoting peer respect. The active involvement of teachers, school nurses, and administrators is essential for successful implementation, as their support leads to a stronger enforcement of FA policies and a deeper commitment to student welfare [20,31].

Health professionals, including pediatricians, school nurses, and other school health professionals, collaborate closely with schools to support children who are victims of FA-related bullying [47,48]. This collaboration is based on a multidisciplinary approach that combines education, prevention, and individualized interventions with the goal of ensuring physical safety and psychological well-being [47].

Evidence consistently highlights that while general school-based anti-bullying programs can reduce bullying rates by around 20% [49], children with FAs remain particularly vulnerable to bullying and require more tailored interventions. Collaboration among families, healthcare providers, and schools is crucial to protect at-risk students and increase awareness of FA-specific risks, which can enhance prevention and intervention efforts [12,31].

#### 3.8.2. Support Mechanisms for Families and Children Affected by FA-Related Bullying

The psychological impact of FA-related bullying extends beyond the child, often affecting their family members. Family-centered counseling, community support groups, and proactive communication with school staff are critical for managing the emotional strain that FA-affected families experience. These resources empower parents to identify signs of bullying and effectively intervene, fostering resilience within the family [11,45]. Counseling programs tailored for children with FAs and their families have demonstrated improvements in quality of life by alleviating feelings of isolation and anxiety [20].

It is important to provide comprehensive support to parents of children with FAs, particularly in relation to access to mental health services. Health professionals should discuss mental health risks with children with FAs and their families and provide information about support services. As much of the bullying of children with FAs occurs by peers in schools [28,39], support services in schools should not only focus on the children involved in the bullying, but also extend to the children’s families. Increased awareness and treatment for the mental health of parents can go a long way towards alleviating some of the difficulties that these families face [34].

For children directly affected by bullying, regular mental health follow-ups are invaluable in addressing long-term effects, such as anxiety, depression, and social withdrawal. Involving mental health professionals provides both children and families with effective coping strategies to manage the psychological impacts associated with FAs [39]. Establishing a support network—including family members, healthcare providers, and school personnel—facilitates social and emotional integration, fostering a culture of well-being and resilience for FA-affected children [32,45].

## 4. Discussion

FA-related bullying is a distinct form of harassment that targets individuals due to their FA, carrying unique characteristics and consequences compared to other forms of bullying. Understanding these differences is essential for developing effective interventions and support systems. FA-related bullying stands out because of its unique interplay of psychological and physical threats, closely tied to the victim’s medical condition. Unlike bullying based on personal characteristics such as physical appearance or ethnicity, FA-related bullying exploits the health vulnerability of children with FAs, introducing an immediate and serious risk to their safety. Perpetrators may use allergens as weapons of intimidation or harm, engaging in actions such as waving, throwing, or contaminating personal belongings with allergenic foods, and even forcing victims into physical contact with allergens. Such behaviors not only inflict emotional distress but can provoke life-threatening allergic reactions, including anaphylaxis [11,27,28,30].

The prevalence of FA-related bullying among children and adolescents with FAs ranges from 17% to 60%, depending on the assessment method and population studied [21,39]. Unlike other forms of bullying, it often involves the intentional use of allergens as tools of intimidation, with behaviors such as deliberate food contamination, physical threats involving allergens, and both physical and psychological harassment [25,28]. This form of bullying is particularly concerning because it incorporates a physical dimension into the power imbalance, making it uniquely hazardous. Studies report that up to 57% of victims have experienced direct contact with allergens during bullying episodes, such as having allergenic substances thrown at them or being forcibly exposed to them [28]. Verbal teasing, social exclusion, and intentional allergen exposure are also common, further compounding psychological harm [14,31,32,33]. These incidents underscore the profound psychological and physical toll bullying imposes on children with FAs and their families.

FA-related bullying’s dual impact on physical and psychological well-being highlights the urgent need for targeted interventions. Routine screening for bullying in pediatric allergy clinics is crucial, given the high prevalence of bullying among children with FAs. Screening allows healthcare providers to detect bullying early and address associated risks before they escalate into severe psychological or physical harm. Consistent screening can significantly lessen the psychological burden on both children and caregivers, promoting a holistic care model that addresses the social dimensions of FAs alongside clinical management [11,26]. Proactive interventions from parents, clinicians, and schools can reduce bullying and improve quality of life (QoL) for affected children [20].

Addressing FA-related bullying requires a multidisciplinary approach, combining education, prevention, and individualized interventions. Healthcare professionals and school staff should collaborate to ensure both physical safety and psychological well-being for affected children. School nurses are pivotal in this effort, as they can develop Individualized Health Care Plans (IHCPs) and Emergency Care Plans (ECPs). These plans, created in collaboration with families, pediatricians, and school staff, include measures to prevent allergen exposure and detailed instructions for emergency responses, such as using epinephrine auto-injectors [47,50,51,52]. School nurses also train teachers, students, and staff on FA awareness, symptom recognition, and emergency response protocols. These structured educational programs foster empathy, reduce stigma, and promote inclusion, significantly decreasing bullying incidents and creating safer school environments [28,30,42,53].

In countries offering free meal programs, several improvements can be implemented to reduce the risk of FA-related bullying. Schools should ensure that allergen-free meals are readily available and clearly labeled. Additionally, providing training to cafeteria staff on allergen management and inclusive meal preparation is critical to avoid accidental exposure and the stigmatization of children with FAs. Establishing allergen-free zones or integrating universal precautionary practices minimizes risks while fostering inclusivity [37,54,55]. Moreover, educating all students about FAs and their consequences through school-wide campaigns promotes empathy and reduces bullying tendencies [47,54,56]. These measures should be complemented by robust anti-bullying initiatives specifically addressing FA-related bullying and emphasizing peer respect [7].

Routine structured inquiries about bullying during clinical assessments enable healthcare providers to create a supportive environment where children and families feel comfortable sharing their experiences [57,58]. School nurses, as first points of contact, can identify physical and emotional signs of bullying, such as headaches, stomachaches, or anxiety, and work with teachers, families, and social workers to implement effective anti-bullying measures [58,59,60]. By identifying these signs and addressing them through coordinated efforts, school nurses can ensure that children with FAs receive timely and appropriate support.

Family involvement is equally critical in order to reinforce strategies at home and foster a comprehensive support system. Research shows that coordinated efforts between families, schools, and healthcare providers reduce the prevalence of FA-related bullying, creating environments where children feel safe, supported, and empowered to manage their condition [50]. Counseling and resilience-building programs help children cope with bullying’s psychological effects, teaching them stress management skills and improving self-esteem [12,61]. Such programs also emphasize the importance of inclusive school cultures, which significantly reduce bullying and improve the psychological well-being of victims [30]. Support must also extend to families, who often experience heightened anxiety due to their child’s vulnerability. Providing resources such as counseling services and FA support groups reduces anxiety and fosters resilience among families, improving overall QoL [33,62]. These groups emphasize that FAs can be effectively managed without significantly impacting daily life, empowering families and instilling confidence in managing the condition [19,61].

Addressing the long-term consequences of FA-related bullying involves regular mental health follow-ups for victims and their families. Clinicians must monitor issues such as anxiety, depression, and social withdrawal, which are common in children who experience bullying [20,39]. Peer education and school-wide anti-bullying campaigns further enhance efforts, fostering empathy and mutual support among students. Collaboration among families, healthcare providers, and educators ensures a comprehensive approach to preventing and managing FA-related bullying [41].

Policymakers also play a crucial role in protecting children with FAs by enacting comprehensive guidelines and legislation. This includes mandatory allergy awareness training for all school staff, the development of individualized allergy management plans (including ECPs), and requiring the presence of epinephrine auto-injectors on school premises [37,55]. Schools should also be mandated to document and report all bullying incidents, including those related to FAs, as part of broader anti-bullying strategies [25,47,56]. Introducing national standards for allergy-friendly school environments and conducting periodic audits ensures compliance with safety standards and fosters inclusivity [25,54].

In conclusion, FA-related bullying represents a severe threat to the well-being of children with FAs. Through multidisciplinary approaches involving education, prevention, and collaboration, schools, healthcare providers, and families can address this issue effectively. These interventions, grounded in inclusivity and proactive prevention, aim to create safer and more supportive environments for children with FA, ultimately reducing bullying and its psychosocial impact while improving QoL for affected children and their families [25,63,64].

## 5. Limitations of the Current Literature

Despite a growing body of research on FA-related bullying, several limitations hinder a comprehensive understanding of its prevalence, characteristics, and impact. One key limitation is the variability in definitions and assessment methods across studies, leading to a wide range in reported bullying prevalence from 17% to 60%, depending on the population and tools used [21,39]. This inconsistency complicates comparisons between studies and limits the generalizability of findings.

Another significant limitation is the lack of longitudinal studies examining the long-term psychological impact of FA-related bullying on children and their families. Although cross-sectional studies provide valuable insights, they cannot capture the evolving nature of bullying experiences and their potential long-term effects on mental health, which include chronic anxiety, depression, and social withdrawal [21,39]. Future research would benefit from longitudinal designs that track outcomes over time, allowing for a better understanding of the lasting consequences of bullying related to FAs.

Moreover, most studies focus primarily on Western, high-income countries, which may not reflect the experiences of children with FAs in diverse cultural and socioeconomic contexts. FA-related bullying and its management strategies may vary based on cultural attitudes towards allergies and bullying behaviors. Expanding research to include low- and middle-income countries would provide a more global perspective on FA-related bullying and help identify cultural factors that may influence bullying behaviors and interventions.

Demographic factors, such as age, gender, and number of FAs, are often inconsistently examined, despite evidence suggesting that these factors significantly affect the likelihood and impact of bullying. For example, older children and those with multiple FAs appear more susceptible to bullying [27,30]. A more nuanced approach that consistently examines these variables would enable targeted interventions and enhance our understanding of specific at-risk groups.

Additionally, there is limited investigation into the role of school policies and staff training in mitigating FA-related bullying. Although some studies highlight the benefits of educational interventions and awareness programs, rigorous evaluations of specific school policies and their effectiveness in reducing bullying are scarce. Future studies could benefit from an in-depth examination of school-based interventions and their long-term impact on reducing FA-related bullying incidents.

Finally, the psychosocial support needs of FA-affected families are underrepresented in the literature. While studies have acknowledged the mental health burden on children with FAs, few explore comprehensive support mechanisms that involve parents and caregivers, who often face heightened anxiety and stress. Future research should aim to evaluate family-centered interventions that empower caregivers to identify and address signs of bullying and support their children effectively.

## 6. Conclusions

FA-related bullying represents a significant and multifaceted challenge for children with FAs, profoundly affecting their mental health, social interactions, and QoL. This review underscores that FA-related bullying is a prevalent issue—with varying rates influenced by demographic factors—that poses distinct risks, as it often involves the use of allergens as tools of harassment. The consequences of this specific form of bullying extend beyond the children affected to include families, who experience increased anxiety, stress, and feelings of helplessness.

Key findings reveal that FA-related bullying is particularly prevalent in school environments, highlighting the need for educational settings to adopt robust anti-bullying measures that address both the physical safety and psychological well-being of children with FAs. School-based programs that promote allergy awareness, empathy, and social inclusivity have shown promise in reducing FA-related bullying and fostering a supportive environment.

Despite these insights, this review identifies critical gaps in the current literature, particularly regarding the need for consistent definitions and assessment methods, the long-term psychological impact on children and families, and the effectiveness of school policies and interventions in diverse cultural and socioeconomic contexts.

Recommendations for future research and policy development include the following:

1. Future studies should employ longitudinal designs to track the enduring impact of FA-related bullying on the mental health and QoL of children and their families.
2. Expanding research to low- and middle-income countries would enhance our understanding of cultural influences on FA-related bullying and assist in identifying global best practices.
3. Consistent examination of demographic factors such as age, gender, and allergy type could enable more targeted interventions.
4. Evaluating school policies and staff training effectiveness is essential for developing evidence-based interventions that can be widely adopted.
5. Family-centered support mechanisms should be investigated to provide comprehensive resources that address the psychological needs of both children and caregivers.

### Call to Action

A collaborative approach involving healthcare providers, educators, policymakers, and families is vital to protect children with FAs from bullying and its associated risks. By implementing evidence-based policies, raising awareness, and providing structured support systems, stakeholders can work together to create a safer, more inclusive environment for FA-affected children, ultimately enhancing their QoL and mental well-being.

## Figures and Tables

**Table 1:** Quality assessment of the reviewed studies according to the Newcastle–Ottawa Scale.

First Author	Year	Study Type	Selection	Comparability	Outcome/Exposure	Total Stars
Abrams [11]	2020	Qualitative (Not scored)	N/A	N/A	N/A	N/A
Annunziato [20]	2014	Longitudinal	3	2	3	8
Bingemann [12]	2020	Cross-sectional	2	1	3	6
Brown [21]	2021	Cross-sectional	3	1	3	7
Cooke [25]	2022	Cross-sectional	3	1	3	7
DunnGalvin [26]	2020	Cross-sectional	3	1	3	7
Fong [27]	2018	Cross-sectional	3	1	2	6
Lieberman [28]	2010	Cross-sectional	2	1	2	5
Merril [29]	2022	Cross-sectional	3	2	3	8
Muraro [30]	2014	Cross-sectional	2	1	2	5
Polloni [31]	2016	Cross-sectional	2	1	3	6
Rocheleau [32]	2020	Cross-sectional	2	1	2	5
Rocheleau [33]	2022	Cross-sectional	3	1	3	7
Rocheleau [34]	2019	Cross-sectional	3	1	3	7
Rocheleau [35]	2019	Cross-sectional	2	1	3	6
Ross [36]	2022	Cross-sectional	3	2	3	8
Ruran [37]	2023	Cross-sectional	3	1	2	6
Sansweet [38]	2024	Cross-sectional	2	1	3	6
Shemesh [39]	2013	Cross-sectional	3	1	3	7
Singh [40]	2003	Cross-sectional	2	1	3	6
Strinnholm [41]	2017	Cross-sectional	3	2	2	7
Torabi [42]	2016	Cross-sectional	2	1	2	5
Tsoumani [43]	2022	Cross-sectional	2	1	3	6
Warren [44]	2016	Cross-sectional	3	1	3	7
Wei [45]	2016	Cross-sectional	3	1	2	6
Yamamoto-Hanada [46]	2015	Cross-sectional	2	1	2	5

N/A: not applicable.

**Table 2:** Bullying characteristics across different countries.

Country	Prevalence (%)	Common Bullying Characteristics	Key Interventions	First Author	Year	Journal
Canada	20%	Repeated bullying (77%), mostly on school grounds perpetrated by classmates, including non-physical acts	Wearing medical ID bracelets, increased education	Torabi [42]	2016	*Paediatr. Child Health*
USA	35–45%	Teasing, threats involving allergens; higher bullying prevalence compared to non-allergic peers	Allergist inquiries, counseling	Bingemann [12]	2020	*J. Allergy Clin. Immunol. Pract.*
Italy	50–60% (relative risk 2×)	Teasing, exclusion in schools	Counseling, structured peer education	Muraro [30]	2014	*J. Allergy Clin. Immunol.*
UK and Ireland	52%	Food-related teasing, frustration, stress due to peanut allergy	Comprehensive public health and psychological programs	Tsoumani [43]	2022	*PLoS ONE*
Australia	42% (general), 23% (allergy-specific)	Bullying involving allergens; allergic reactions during bullying episodes	Allergy education, school-based programs	Fong [27]	2018	*Pediatric Allergy Immunology*
Japan	2.8%	Minimal acknowledgment of bullying; anxiety over adverse events	Community and caregiver education	Yamamoto-Hanada [46]	2015	*Pediatric Allergy Immunology*
Sweden	5%	Hypersensitivity-related lower HRQL among children	Enhanced food labeling, support programs	Strinnholm [41]	2017	*Clin. Transl. Allergy*
Canada	19% (children with FA)	Social stigma, lack of awareness among school staff	Training for teachers, school-wide allergy education	Ross [36]	2020	*Allergy Asthma & Clin. Immunology*
Italy	N/A	Teachers underestimate bullying risk and relational difficulties	Teacher training on FA awareness and management	Polloni [31]	2016	*Pediatric Allergy Immunology*
Sweden	N/A	Emotional distress, isolation due to food restrictions	Awareness campaigns, targeted support programs	Strinnholm [41]	2017	*Clin. Transl. Allergy*
USA	31%	Physical and verbal bullying, allergen use as harassment method	Education plans, parental and peer support	Cooke [25]	2022	*Journal of Pediatric Psychology*
Taiwan	N/A	Chronic disease-linked bullying, particularly among children with allergies and asthma	Sensitivity training for school staff	Wei [45]	2016	*Journal of School Nursing*
USA	20% (FA-specific bullying)	FA-related bullying mainly from classmates, linked to lack of school allergen policies	Policy changes, parent–school collaboration	Brown [21]	2021	*Ann. Allergy Asthma Immunol.*
USA	35–45%	FA-related bullying, often involving allergens being thrown	School nurse involvement, increasing teacher awareness	Ruran [37]	2023	*J. Allergy Clin. Immunol. Pract.*
USA	24%	Allergens used as tools for harassment; teasing often repeated	Educating peers and school communities	Lieberman [28]	2010	*Ann. Allergy Asthma Immunol.*

## Data Availability

No new data were created or analyzed in this study. Data sharing is not applicable to this article.
